# Osmolytes Modulate Photoactivation of Phytochrome: Probing Protein Hydration

**DOI:** 10.3390/molecules28166121

**Published:** 2023-08-18

**Authors:** Jens Balke, Paula Díaz Gutiérrez, Timm Rafaluk-Mohr, Jonas Proksch, Beate Koksch, Ulrike Alexiev

**Affiliations:** 1 Department of Physics, Freie Universität Berlin, Arnimallee 14, 14195 Berlin, Germany; 2 Department of Biology, Chemistry, Pharmacy, Institute of Chemistry and Biochemistry–Organic Chemistry, Freie Universität Berlin, Takustr. 3, 14195 Berlin, Germany; jonas.proksch@fu-berlin.de (J.P.); beate.koksch@fu-berlin.de (B.K.)

**Keywords:** phytochrome, Cph1, Pr, Pfr, hydration, osmotic stress

## Abstract

Phytochromes are bistable red/far-red light-responsive photoreceptor proteins found in plants, fungi, and bacteria. Light-activation of the prototypical phytochrome Cph1 from the cyanobacterium *Synechocystis* sp. PCC 6803 allows photoisomerization of the bilin chromophore in the photosensory module and a subsequent series of intermediate states leading from the red absorbing Pr to the far-red-absorbing Pfr state. We show here via osmotic and hydrostatic pressure-based measurements that hydration of the photoreceptor modulates the photoconversion kinetics in a controlled manner. While small osmolytes like sucrose accelerate Pfr formation, large polymer osmolytes like PEG 4000 delay the formation of Pfr. Thus, we hypothesize that an influx of mobile water into the photosensory domain is necessary for proceeding to the Pfr state. We suggest that protein hydration changes are a molecular event that occurs during photoconversion to Pfr, in addition to light activation, ultrafast electric field changes, photoisomerization, proton release and uptake, and the major conformational change leading to signal transmission, or simultaneously with one of these events. Moreover, we discuss this finding in light of the use of Cph1-PGP as a hydration sensor, e.g., for the characterization of novel hydrogel biomaterials.

## 1. Introduction

**Figure 1:**
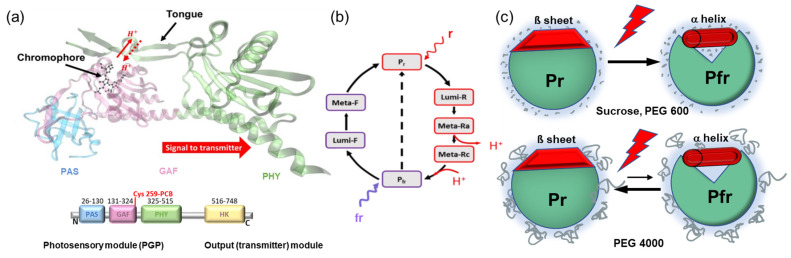
(**a**) Structural model of Cph1-PGP (PDB: 2VEA). PAS (P, blue), GAF (G, light magenta), and PHY (P, green) denote the domains in the photosensory module (PGP); the amino acid numbers are given in the scheme below together with the output module for signal transduction (yellow). The PCB chromophore is bound to cysteine in position 256. The structural motif, called the tongue, connects the PAS domain with the chromophore binding pocket and changes its conformation between the β-sheet in Pr and the α-helix in Pfr. (**b**) Cph1 photocycle with red (r) and far red (fr) light excitation. Upon Pr excitation photoisomerization (Pr to Lumi R), chromophore relaxation, transient deprotonation, and large conformational changes (Meta Ra to Meta Rc to Pfr) take place. (**c**) Potential osmotic stress effects on the Pr-to-Pfr photoreaction are schematically shown. Illustrated is a situation where water changes (water influx) play a role in photoconversion. The photoreaction is shifted either forward or backward, depending on the osmolyte size. Small osmolytes penetrate the protein, thus favoring Pfr formation. Large polymer osmolytes like PEG 4000 are excluded from the protein to withdraw water, thus disfavoring Pfr formation.

Phytochrome photoreceptors were first discovered in plants, and later found in bacteria, fungi, and algae [1,2,3]. In plants, phytochromes control many developmental processes, such as seed germination, de-etiolation, and flowering [1]. The discovery of the bacterial phytochrome Cph1 from the cyanobacterium *Synechocystis* sp. PCC 6803 points to the prokaryotic origin of these photoreceptors [3,4,5]. These so-called prototypical phytochromes act as photochemical switches that interconvert between stable red (Pr)- and metastable far-red (Pfr)-absorbing states induced by photoisomerization of the bilin chromophore after light activation [6,7,8]. The bilin chromophore, phycocyanobilin (PCB) in the case of Cph1, is bound to a cysteine in position 259 in the GAF (cGMP phosphodiesterase/adenyl cyclase/FhlA) domain of Cph1, which is flanked by a PAS (Per/Arnt/Sim) and PHY (phytochrome-specific) domain. Together, the PAS, GAF, and PHY domains (PGP) constitute the photosensory module (Figure 1a). The PCB chromophore deprotonates transiently during the Pr→Pfr photoconversion in association with extensive global structural changes required for signal transmission [9,10] (Figure 1a,b). In prototypical phytochromes, the transition to Pfr is associated with a restructuring of the tongue in the PHY domain (Figure 1a) from an antiparallel β-sheet to an α-helix [9]. This secondary structure transition is thought to be instrumental in coupling the structural changes of the chromophore binding pocket to the large global conformational/structural changes that are postulated to establish the interaction between the photosensory module and the C-terminal transmitter (output) module (Figure 1a). Phytochromes usually contain C-terminal histidine kinase domains that show light-regulated protein kinase activity. This activity was first reported for Cph1 [11]. All these events eventually lead to the activation of the physiological signal.

A growing number of studies on phytochrome have revealed that proton translocation has a crucial role in the coupling of chromophore and protein conformational changes [10,12,13,14,15,16,17,18]. Proton translocation depends on hydrogen bond networks and structural water molecules within proteins. The dynamics of water molecules at the soluble protein-water interface, however, is only 3–5 times slower than in bulk water, facilitating protein conformational changes [19]. Proton translocation and the involvement of water, as well as water-assisted conformational changes, have been investigated and proposed for a number of photoreceptor proteins. This includes both soluble and membrane proteins.

In membrane (photoreceptor) proteins, proton translocation—in particular proton uptake—is often accompanied by water uptake, as revealed by crystallographic and spectroscopic studies of membrane proton pumps such as bacteriorhodopsin [20,21,22,23] or cytochrome c oxidase [24]. For the transmembrane photoreceptor visual rhodopsin, a G-protein-coupled receptor, it was recently shown that mobile water influx into the protein core is necessary for receptor activation [25].

In soluble LOV (Light, Oxygen, and Voltage) photoreceptors, for example, it was found that water plays a major role in the dark reversion kinetics [26]. Faster dark reversion kinetics in bacterial LOV domain proteins were reported to depend on partial dehydration and were interpreted as an effect facilitated by a trapped conformational intermediate [27]. Other studies on plant LOV photoreceptors showed that the effect of dehydration on the kinetics was similar to the effect of lowering the temperature [28]. The data were interpreted as an effect of dehydration on protein structural rigidity. Furthermore, a buried water cluster acting as a proton acceptor in the rate-limiting step of dark state regeneration has been proposed by other authors as an explanation for the hydration dependence [29].

The role of hydration in phytochrome photoconversion was recognized very early in studies of the germination process of dry seeds [30,31,32]. It was shown that dehydration had an important effect on the photoconversion/-reversibility of phytochrome, as inferred directly from the absorbance spectra of purified plant phytochrome [30] or indirectly via seed germinability as an indicator of Pr→Pfr photoconversion [31]. From the thermodynamic data, it was concluded that water molecules bind very strongly to phytochrome at hydration levels below 8% (water content in seeds), a regime in which no measurable Pr→Pfr photoconversion was detected [31]. This behavior was attributed to conformational changes constraint by the very tightly bound water molecules but otherwise occurring in the reaction from the transient Lumi-R intermediate to Pfr [31]. Hydrogen/deuterium exchange rate analyses of plant phytochrome B showed that the local refolding in the tongue region upon Pr→Pfr photoconversion is accompanied by higher exchange rates in the helical state, indicating a larger mobility as well as less shielding from bulk water for this region in Pfr [33]. For Cph1, partial dehydration by ammonium sulfate precipitation in NMR experiments resulted in a decrease in chromophore mobility [34]. In cyanobacteriochromes, which possess only the GAF domain that fulfills both the covalent bilin chromophore binding and the photochemistry, a hydration increase in the chromophore binding pocket of AnPixJg2 was observed upon photoconversion [35]. These results suggest that dehydration may extend to the protein interior.

Inspired by the osmotic stress method [36] used to measure the water influx upon activation of the photoreceptor rhodopsin [25], we here test the hypothesis that osmolytes can also affect the photoreaction from the Pr to the Pfr state in the photoreceptor phytochrome. Osmotic stress from lowering water activity via polymer osmolytes of different sizes, like polyethylene glycol (PEG) [37], could probe the hydration sensitivity of the Pr→Pfr reaction.

By analogy to the results of visual rhodopsin activation by water [25], we show here the possible effect of small osmolytes and polymer osmolytes of varying molecular weight on the putative opening of a cleft that allows water to access the protein interior (Figure 1c). Our results indicate that both molecular osmolytes and polymeric low molecular weight osmolytes can slightly accelerate Pfr formation, suggesting hydration (water influx) in the photosensor. As the size of the polymer osmolyte increases, however, the kinetics of Pr→Pfr slow down. In order to account for possible macromolecular crowding effects by the higher molecular weight PEG polymers [38,39], we mimicked this non-specific, physical interference effect by using the inert branched polysaccharide Ficoll 400 and compared the kinetics with those of monomeric sucrose. Crowding might impact protein structure, folding, or conformational stability by, e.g., stabilizing the closed protein state [40]. In addition, possible viscosity effects were ruled out by using a PEG 1000 solution that was adjusted to a solvent viscosity similar to sucrose. The fact that sucrose and Ficoll 400 had similar effects on Cph1 photoconversion kinetics makes molecular crowding effects less likely. Thus, changing water activity very likely affects phytochrome photoactivation and its physiological function in the crowded cellular environment.

## 2. Results

### 2.1. Spectroscopic Characterization and Pr→Pfr Photoconversion

**Figure 2:**
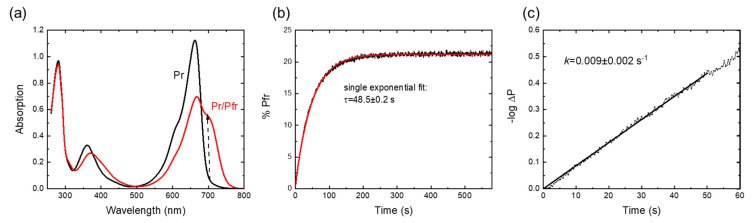
Photoconversion kinetics of Cph1-PGP. (**a**) Absorbance spectra after far-red irradiation (black) and red irradiation (red). (**b**) Photoconversion kinetics after 20 s of LED irradiation with λ_LED_ = 680 nm. The time trace can be fitted with a single exponential, allowing approximation by the initial slope method as shown in (**c**). The time constant of the single exponential fit is given. (**c**) Determination of the photoconversion rate constant using the initial slope of the –logΔ*P* vs. *t* curves with Δ*P* = ([Pfr]_t_ − [Pfr]_∞_)/([Pfr]_0_ − [Pfr]_∞_). Conditions: 300 mM NaCl, 50 mM Tris, pH 7.8, RT (23 ± 1 °C).

The UV-vis absorbance spectra of the red and far-red light-illuminated Cph1-PGP sample are shown in Figure 2a and display spectral features in agreement with earlier work [5,14,41]. Pr→Pfr photoconversion kinetics were recorded after illumination with 20 s of far red light and are shown in Figure 2b. Analysis of the initial slopes yields the photoconversion rate constant (Figure 2c) [42]. For Cph1 in buffer solution, the obtained rate constant of 0.01 ± 0.003 s^−1^ agrees excellently with literature values of 0.01 s^−1^ [43]. The recording of these simple Pr→Pfr photoconversion kinetics was chosen to directly visualize the potential effects of osmolytes.

### 2.2. Osmolyte Effects on Pr→Pfr Photoconversion

**Figure 3:**
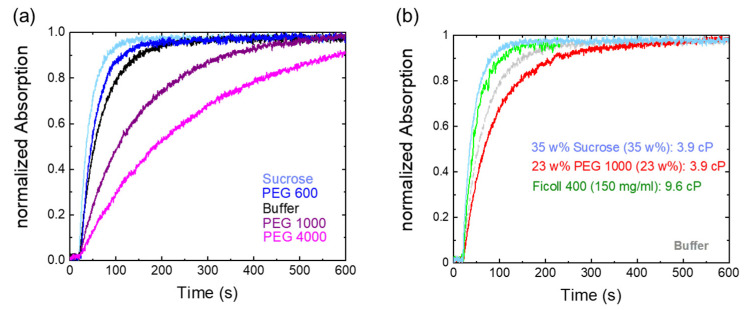
Osmolytes and macromolecular crowding effects on Cph1-PGP photoconversion kinetics. (**a**) Different osmolytes affect Pr to Pfr kinetics; low molecular weight PEG (35 w% PEG 600) and 35 w% sucrose slightly accelerate photoconversion kinetics, while large PEGs (35 w% PEG 1000, 35 w% PEG 4000) slow down photocycle kinetics. (**b**) Increased viscosity is not the cause of delayed kinetics, as shown by the comparison of sucrose and PEG 1000 at similar viscosities of 3.9 mPas. For comparison with the effect of a macromolecular crowder, the time trace in a polymeric sucrose (Ficoll 400) solution is also shown. Gray indicates the time trace in buffer.

Figure 3 shows the Pr→Pfr photoconversion kinetics in the presence of different osmolytes. The osmotic stress of lowering water activity has been used to measure the interaction energies between large molecular surfaces, such as proteins, and the change in the number of surface-associated water molecules [37]. We use here the water-soluble polyethylene glycol (PEG) polymers, as relatively high osmotic pressures (Π) of several MPa can be achieved [36]. PEG is able to bind water nearly equivalent to its own weight and thus removes the water molecules from being active [37].

Surprisingly, and in astonishing agreement with the behavior of visual rhodopsin activation [25], Pfr photoconversion was facilitated by sucrose and low-molecular-weight PEG. Higher molecular weight PEG molecules increasingly reduce the photoconversion. The corresponding rate constants are summarized in Table 1. The reported kinetic constants in pure buffer, sucrose, and PEG 600, however, are quite similar when taking errors into account. The statistical analysis was done using a one-way ANOVA test. No significant differences in mean rate values were found between buffer and sucrose, respectively, PEG 600, with a significance level of 0.05. Nevertheless, there is a trend towards faster kinetics (Figure 3a). Moreover, at the same weight percentage, the photoconversion kinetics in PEG 1000 are clearly slower than in PEG 600 solution and in buffer alone.

To rule out that viscosity effects play a role in the differential action of small and high molecular weight osmolytes, we recorded the photoconversion kinetics for sucrose and PEG 1000 at an identical solvent viscosity of 3.9 mPas (Figure 3b). Obviously, and although the concentration of PEG 1000 was reduced compared to the experiments shown in Figure 3a to achieve similar viscosity values as in sucrose, the slower photoconversion kinetics compared to buffer solution remain. This implies that the slower photoconversion kinetics of higher molecular weight PEGs compared to sucrose are not due to the different osmolytes having different viscosities. To mimic possible macromolecular crowding effects, we also measured the kinetics of Ficoll 400, a polymeric branched sucrose (Figure 3b). Photoconversion kinetics in Ficoll 400 are as fast within error as in monomeric sucrose solutions, even though the viscosity is slightly higher.

### 2.3. Determination of the Apparent Number of Water Molecules That Penetrate into Cph1 upon Photoconversion

Thermodynamics relations connect the hydrostatic pressure to the work of transporting water in or out of the protein interior, the external hydrostatic pressure, and the change in hydrated volume. From the change in hydrated volume Δ*V*° and using the equilibrium constant *K*, derived from the rate constants, we can obtain the number of apparent water molecules *N*_w_ (Equation (3)) [25,37].

**Figure 4:**
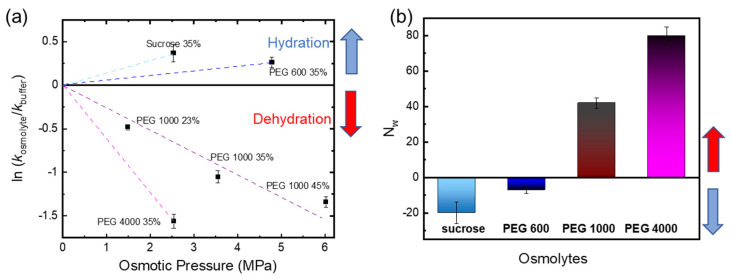
Estimation of the number of water molecules taken up or released from red light-irradiated Cph1-PGP for polymer (PEG) osmolytes and sucrose according to Equation (3). (**a**) ln *K* plotted versus osmotic pressure for different sizes of PEG osmolytes, sucrose, and buffer based on results in Figure 3. The dashed lines guide the eye. For PEG 1000, the values for the different concentrations follow the dashed line, indicating the same apparent change in water molecules based on the slope according to Equation (3). (**b**) Apparent number (*N*_W_) of water molecules entering or leaving the Cph1 protein interior based on Equation (3).

Figure 4 shows a negative slope for high molecular mass PEG osmolytes (PEG 1000 and PEG 4000), while a positive slope was found for small PEG (PEG 600) and sucrose. Positive values of work indicate water uptake, while negative values are indicative of water release. Thus, a negative slope would be due to dehydration, a positive slope due to hydration in the framework of the osmotic stress method. Based on these data, we hypothesize that large osmolytes dehydrate the phytochrome because they cannot penetrate inside the protein but draw water and reduce water activity. The apparent number of water molecules is given in Figure 4b. Table 2 summarizes the values for work, ln *K*, and the apparent number of water molecules.

### 2.4. Hydrogel Effects on Pr→Pfr Photoconversion

The sensitivity of Cph1-PGP photoconversion to osmolytes and, as indicated, to water activity (hydration) may allow Cph1 to be used as a water-state sensor at the nanoscale. In particular, bio-inspired hydrogels have received a lot of attention recently [44,45,46]. Hydrogels are three-dimensional networks of cross-linked hydrophilic polymer chains. Similar to the PEG macromolecules we used here as macromolecular osmolytes, hydrogels can bind large amounts of water. This water inside hydrogels exists as bound, non-freezable water, as freezable bound water, and as free water like in aqueous buffer solutions [46].

**Figure 5:**
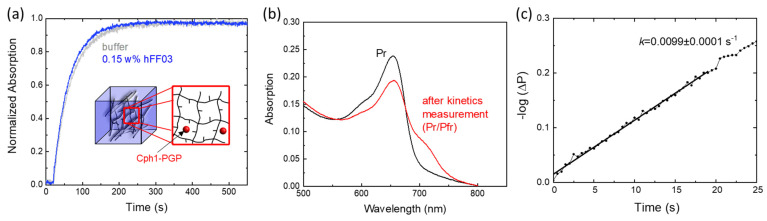
Photoconversion kinetics of Cph1-PGP in the peptide hydrogel hFF03. (**a**) Photoconversion kinetics in hFF03 and in buffer (see Figure 2b,c). (**b**) Absorbance spectra after far-red irradiation (black) and kinetics recording (red). (**c**) Rate determination from the initial slope. The photoconversion rate is given in the figure. Conditions as in Figure 2 and Table 1.

To test how the water inside a hydrogel affects Cph1-PGP, we measured the Cph1-PGP photoconversion kinetics in the peptide hydrogel hFF03. hFF03 is a coiled coil-based peptide that self-assembles into a 3D-α-helical fibril network and functions as a self-supporting hydrogel [45]. Within the three-dimensional network of hFF03, Cph1-PGP should be affected by the different types of water. As shown in our experiments with PEG, we expect that a high fraction of bound water will slow down the kinetics of photoconversion, while a high fraction of free water should lead to kinetics close to those in buffer solution. Figure 5a presents the recorded photoconversion kinetics from Pr to Pfr in 0.15 wt% hFF03 and the corresponding absorbance spectra before and after recording the kinetics (Figure 5b). The photoconversion kinetics are similar to those observed in buffer (Figure 5a,b, Table 1).

## 3. Discussion

In this study, we investigated the effect of osmolytes—small-molecule osmolytes such as sucrose and large macromolecular PEG osmolytes with different molecular weights—on the photoconversion kinetics of phytochrome Cph1-PGP. The addition of PEG molecules of different molecular weight to Cph1-PGP solutions decreases the photoconversion rate in a size-dependent manner. Surprisingly, small osmolytes, including PEG 600, slightly accelerate the photoconversion kinetics, although experimental errors must be taken into account (Figure 3a). The results can be interpreted on the basis that PEG acts indirectly by means of water activity (osmotic effect) [37]. This interpretation is supported by previous observations of the effects of dehydration on plant phytochrome in seeds. There, using percent germination as a readout, a slower relative rate of photoconversion from Pr to Pfr was estimated at low moisture content when seeds were exposed to red light for different lengths of time [31]. Several other studies also reported the effects of dehydration on plant phytochromes [30,47]. To account for possible macromolecular crowding and viscosity effects that might influence tongue refolding, we also measured photoconversion kinetics in Ficoll 400, a sucrose polymer that acts as a macromolecular crowder, and in PEG 1000 solution of the same viscosity as that of 35 wt% sucrose. In the former case, similar or slightly faster photoconversion kinetics were measured than in buffer solution, while photoconversion in PEG 1000 at the same viscosity as in 35 wt% sucrose was still significantly slower than in buffer (Figure 3b).

The thermodynamic relations for the osmotic effect on phytochrome photoconversion yield an increase in the number of water molecules connected with the Pr→Pfr photoreaction, i.e., water influx in the protein interior. Large PEG polymers oppose this effect by excluding/withdrawing water molecules from Cph1. This effect might be transient and related to the restructuring of the tongue in the PHY domain (Figure 1a) from an antiparallel β-sheet to an α-helix upon photoconversion. So far, however, only the crystal structure of the Pr state of Cph1-PGP is available [48]. Evidence for a more hydrated tongue in its α-helical conformation comes from experiments with *Arabidopsis* plant phytochrome B, which, like Cph1, also contains PCB as a chromophore [33]. Hydrogen/deuterium exchange measurements of the phytochrome amide backbone reveal a higher hydration of the α-helical tongue conformation [33]. Moreover, studies on the removal of polar residues in the chromophore binding pocket of phytochrome indicated a decrease in water content [18,49] and suggested a role in increasing phytochrome autofluorescence while decreasing the probability for Pr→Pfr photoconversion [41,49].

A closer look at recent osmotic stress measurements using the visual photoreceptor rhodopsin [25] reveals similarities with our data. The ln *K* vs. osmotic pressure plots of visual rhodopsin strongly resemble those of phytochrome (Figure 4a). In analogy, we hypothesize that a cavity for water influx opens in Cph1 upon light activation, into which small osmolytes can penetrate and carry water molecules, thus accelerating Pfr formation in Cph1. We further hypothesize that the conversion of Pr to Pfr requires water influx/hydration of Cph1. Taken together, our analysis provides evidence that osmolytes act on phytochrome photoconversion via changes in water activity. This finding will guide further studies to link the early dehydration experiments in plant seeds [30,31] and our hypothesis about the water influx/hydration of Cph1. We anticipate that crystal structures of the Pfr state and molecular dynamics simulations will shed more light on this molecular event in the future. Moreover, time-resolved photocycle measurements are currently being performed to correlate the kinetics of the photocycle intermediates with the observed osmolyte effects and proton release/uptake kinetics.

From an application point of view, we propose to exploit the sensitivity of Cph1 photoconversion to water activity (hydration) and use Cph1 as a nanoscale hydration sensor in hydrogels. Hydrogels are widely used for biomedical applications [50]. In an initial experiment, we tested the water structure within a novel peptide hydrogel, hFF03 [45] (Figure 5). This experiment, using Cph1 as a nanosensor, corresponds to the length scale of water states relevant to the molecular function of proteins. We envision that our photo/hydration sensor can bridge the gap that exists in characterizing novel hydrogels and the state of water using established structural/spectroscopical [46] as well as macro-and micro-rheological [45] techniques and the less explored nanoscale.

## 4. Materials and Methods

### 4.1. Phytochrome Expression and Purification

The photosensory module of wild-type Cph1 (residues 1-514 with a C-terminal His6-tag, Cph1-PGP) was expressed in *E. coli*, the cells lysed, and the soluble fraction purified by Ni^2+^-affinity and size-exclusion chromatography [48]. Apo-protein assembly with PCB was done according to established protocols [5]. The extinction coefficient of the Pr state (pH 7.8) at the maximum wavelength (λ_max_ = 660 nm) with 85,000 M^−1^ cm^−1^ was used for concentration determination [5].

### 4.2. Synthesis of Peptide hFF03

The peptide hFF03 was synthesized according to established procedures [45] with a microwave-assisted automated synthesizer Liberty Blue (CEM, Matthews, NC, USA) using standard fluorenylmethoxycarbonyl (Fmoc) chemistry with diisopropylcarbidiimide (DIC) as the activating agent and ethyl 2-cyano-2-(hydroxyamino)acetate (Oxyma Pure) as an additive. Fmoc deprotection was achieved using piperidine (20% (*v*/*v*) in DMF). The synthesis was performed on a 0.1 mmol scale with Fmoc-Leu-NovaSyn TGA resin (0.19 mmol/g loading). A 5-fold excess of Fmoc amino acid (Carbolution) was used in each coupling. Cleavage from the resin was achieved by treatment with a mixture of trifluoroacetic acid (TFA), triisopropylsilane (TIPS), and water (95:2.5:2.5 (*v*/*v*); 10 mL/g of resin) for 2 h at room temperature. After ether precipitation, the crude peptide was dried by lyophilization and purified using reversed-phase HPLC. Afterwards, the inevitably obtained trifluoroacetate counterions were exchanged for chloride according to literature protocols [51]. The peptide was dissolved in water at 0.5 mM and HCl added to give a final concentration of 7.5 mM. After one minute of stirring, it was lyophilized, and the procedure was repeated a total of five times.

### 4.3. Hydrogel Preparation

A peptide stock solution was prepared in hexafluoroisopropanol (HFIP), and the concentration was determined by UV spectroscopy at 320 nm via N-terminal aminobenzoic acid and a calibration curve [45]. The required amounts of this solution were transferred, and the solvent evaporated in a stream of nitrogen. The resulting peptide film was dissolved in Dulbecco’s phosphate buffered saline (DPBS, *w*/*o* Ca^2+^, Mg^2+^, and Sigma) at a concentration of 0.15 wt% (0.5 mM), vortexed for ten seconds, gently mixed by pipetting, and again vortexed for ten seconds. After 2 and 5 h, the solution was mixed again carefully by pipetting to form a homogeneous hydrogel. The hydrogel was used for Cph1 experiments the following day.

### 4.4. UV-Vis Spectroscopy and Data Analysis

UV-vis absorbance spectra were measured with a Shimadzu UV2450 spectrometer (Shimadzu, Kyoto, Japan).

The CphI-PGP sample in the Pr state in the different osmolyte solvents was illuminated for 20 s in a 90° geometry with a red LED (λ_LED_ = 680 nm, FWHM= 26 nm, Conrad Electronics, operated at 0.55 mW/cm^2^) to initiate photoconversion, and the absorption transient was recorded for 600 s. The Pr→Pfr photoconversion was monitored at λ_Pfr,max_ = 700 nm [10] to measure the increase of the Pfr fraction. The optical path length of the quartz cuvette was 0.3 cm. Absorbance spectra of phytochrome were taken in the initial Pr state (after fr radiation) and after absorption transient measurements, respectively. The photoconversion rate constant of Pfr state *k* was determined by extrapolating the initial slope of the resulting photoconversion and using Butler’s equation:

*k* · *t* = −logΔ*P* (1)

where Δ*P* is the proportion of phytochrome converted, calculated as follows:

Δ*P* = ([Pfr]_t_ − [Pfr]_∞_)/([Pfr]_0_ − [Pfr]_∞_). (2)

[Pfr]_t_ was estimated by proportionally subtracting the absorbance of the spectrum of Pr from the absorbance transient following irradiation [42,43].

From the change in hydrated volume Δ*V*° and using the equilibrium constant *K*, derived from the rate constants, we can derive the number of water molecules *N_w_* with *V_w_* being the standard change in excess (partial) water volume of the initial and final states [25,37]. We used *K* = *k_osmolyte_*/*k_buffer_*, leading to the following equation:

(3) $$\ln(\frac{k_{osmolyte}}{k_{buffer}})=-\frac{N_{w}V_{w}}{RT}\Pi ,$$

allowing us to calculate the apparent water molecules involved in the photoconversion for each osmolyte solution, considering *V_w_* = 0.018 L/mol, *R* = 0.00831 MPa·L/(mol·K), and *T* = 296.15 K. *R* and *T* have their usual meanings. The work of transporting water into or out of the protein interior is calculated by *w* = −Π*N_w_V_w_* = −ΠΔ*V*° [37].

## Figures and Tables

**Table 1:** Photoconversion rates in different size PEG osmolytes, sucrose, and pure buffer solutions (n = 3 independent experiments; standard deviation is given). Conditions as in Figure 2. The concentration of the solutes is given in weight percent unless stated otherwise.

Solvent	*k* (s^−1^)
Pure buffer	0.010 ± 0.003
Sucrose 35%	0.014 ± 0.004
PEG 600 35%	0.013 ± 0.003
PEG 1000 23%	0.0062 ± 0.0004
PEG 1000 35%	0.0035 ± 0.0002
PEG 1000 45%	0.0026 ± 0.0001
PEG 4000 35%	0.0021 ± 0.0001
Ficoll 400 (150 mg/mL)	0.013 ± 0.001

**Table 2:** The number of water molecules *N*_w_ taken up or released, the increase or decrease in the standard change in excess water volume, and the work of transporting water molecules in or out of the protein interior upon Cph1-PGP Pr to Pfr photoconversion are given according to Equation (3). The concentration of the different osmolytes is expressed in weight percent.

Osmolyte	Δ*N_w_*	Δ*V*° (L mol^−1^)	*w* (kJ mol^−1^)
Sucrose 35%	−20 ± 6	−0.30 ± 0.08	0.8 ± 0.3
PEG 600 35%	−7 ± 2	−0.10 ± 0.02	0.5 ± 0.2
PEG 1000 23%	44 ± 3	0.80 ± 0.05	−1.20 ± 0.08
PEG 1000 35%	40 ± 3	0.70 ± 0.05	−1.9 ± 0.2
PEG 4000 35%	80 ± 5	1.50 ± 0.08	−3.8 ± 0.2

## Data Availability

Data is available from corresponding author on request.
